# Correlation Between Postoperative Health-Related Quality of Life and Care Needs of Oral Cancer Patients

**DOI:** 10.1097/NCC.0000000000000677

**Published:** 2018-11-15

**Authors:** Tze-Fang Wang, Yu-Jie Li, Lee-Chen Chen, Chyuan Chou, Su-Chen Yang

**Affiliations:** Author Affiliations: School of Nursing, National Yang Ming University, Taipei (Ms Wang and Mr Li); Department of Nursing, Far Eastern Memorial Hospital, New Taipei (Mss Chen and Yang); and Excellent Dental Center, Taipei (Dr Chou), Taiwan.

**Keywords:** care needs, CNQ-SF, EORCQOL-H &N35, health-related quality of life, oral cancer, Taiwan

## Abstract

Oral cancer is the fifth most common form of cancer in Taiwan in terms of incidence and death rate and results in at least 2700 deaths each year.

**Objective:**

The aims of this study were to assess the postoperative health-related quality of life (QOL) and care needs of oral cancer patients comprehensively and to evaluate the correlation between health-related QOL and care needs.

**Interventions/Methods:**

This cross-sectional study enrolled 126 oral cancer patients who had received surgical treatment within the previous 2 years and were without cognitive impairment. Each patient completed a demographic questionnaire, the European Organisation for Research and Treatment of Cancer Head and Neck Cancer Quality of Life Scale, and the Short-Form Cancer Needs Questionnaire.

**Results:**

Female patients and patients receiving 3 or more chemotherapy treatments were significantly associated with increased Short-Form Cancer Needs Questionnaire scores (higher level of care needs) (*β* = 0.177 and 28.49, both *P* < .05) and patients receiving 3 or more chemotherapy treatments were significantly associated with increased Head and Neck Cancer Quality of Life Scale scores (higher level of symptoms and problems) (*β* = 27.77, *P* = .007). Results of stepwise multiple linear regression analysis indicated that 4 oral cancer–related symptoms and problems, “trouble with social contacts,” “swallowing problems,” “teeth problems,” and “feeling ill,” were significantly associated with higher care needs in oral cancer patients (all *P* ≤ .05).

**Conclusion:**

A significant correlation exists between health-related QOL and care needs.

**Implications for Practice:**

Using a valid health-related QOL scale may help nurses determine their perceived physical and psychological care needs.

Oral cancer is reported by the World Health Organization to have a higher incidence in developing nations than in developed nations and to occur more commonly in men than in women.^1^ It has a higher incidence among both men and women in southern Asia than in other parts of the world. India, Thailand, and China, in that order, are reported to have the highest age-standardized incidence rates in the world, which may be due to the joint influence of increasing contact with risk factors and demographic aging.^2^ Oral cancer ranks fifth among the 10 most common forms of cancer in Taiwan in incidence and death rate and ranks fourth among the most common cancers of men in incidence and death rate.^3^ The average age of death in individuals with oral cancer can be about 10 years earlier than that in those with other cancers, and more than 2700 persons are estimated to die of oral cancer each year in Taiwan.^3^

Treatment of oral cancer applies various methods according to the stage of the cancer as determined by clinical diagnosis. The main treatment methods include surgical removal, either alone or in combination with other methods, local radiation therapy, and chemotherapy. In Taiwan, the major type of oral cancer is squamous cell carcinoma. Because 92% of squamous cell carcinoma cases consist of well-differentiated or moderately differentiated cancer, and tissue of this type is not very sensitive to radiation, surgical removal is currently the most common first-line treatment in Taiwan. Surgery may be supplemented subsequently with other treatment methods as necessary.^4^

The surgical treatment of oral cancer is tumor removal with or without neck lymph node dissection. By removing the primary tumor and neck lymph nodes at the same time, this approach can prevent distant metastasis from the buccal or lingual lymph nodes of the neck. However, this surgery may impair the function and appearance of a patient's face, neck, or mandible, which negatively affects the patient's postoperative body image and ability to open the mouth, swallow, chew, and talk.^5–10^ Patients' quality of life (QOL) will usually deteriorate significantly with surgical treatment. Advanced tumors require extensive surgical resection involving flap reconstruction, neck dissection, and postoperative radiation, which are associated with poor QOL outcomes.^11^

Needs are based on an individual's subjective values. When individuals experience various problems or events, they feel that they can resolve or improve these situations but may be unable to access appropriate resources and effective methods of easy resolution, giving rise to needs.^12^ Research on patients with cancer, AIDS, and heart disease has verified that correlations exist between health-related QOL (HRQOL) and care needs.^13–16^ Among patients with head and neck cancer, low levels of satisfaction with QOL are shown to create relatively high needs.^17^ Molassiotis et al^15^ studied the associations between care needs and QOL in patients with multifocal osteomyelitis and found that QOL was a significant predictive factor for the level of unmet needs. As such, performing an in-depth assessment of care needs may help to identify unmet care needs that reflect an individual's specific characteristics and help caregivers provide appropriate medical care or social support.

We hypothesized that a significant correlation exists between HRQOL and care needs of oral cancer patients. Therefore, this study aimed to assess HRQOL and care needs of postoperative oral cancer patients comprehensively, to identify factors that may be associated with HRQOL and care needs, and to evaluate the correlation between HRQOL and care needs.

## Methods

### Participants

This study used a prospective cross-sectional research design and used purposeful sampling to select subjects from a single medical center in northern Taiwan that provides surgical treatment for oral cancer. Purposeful sampling was performed to increase the likelihood that subjects met acceptance criteria and to reduce uneven distribution of subjects' characteristics and bias. The research subjects were postoperative oral cancer patients in the surgical and outpatient departments of Far Eastern Memorial Hospital from February to May 2016 and were invited to participate in the study by staff members of the 2 departments. Referred patients were enrolled if they met the inclusion criteria, were willing to participate in this study, and signed informed consent forms. Inclusion criteria were patients with a physician's diagnosis of oral cancer who had received surgical treatment within the previous 2 years and were fully alert and conscious and able to communicate in Mandarin or Taiwanese (Minnan). Exclusion criteria were patients who could not understand the research scale and questionnaire after receiving an explanation, including persons with impaired cognitive function, dementia, and intellectual disability. In addition, juveniles, convicts, indigenous citizens, pregnant women, and persons with physical and mental disabilities were excluded. Research codes were used to identify the subjects during the study period, and subjects' personal information was not disclosed. The subjects had the right to verbally notify the researchers at any time of their wish to terminate their participation. The subjects were asked either to fill out the questionnaire and scale themselves or to allow a single trained interviewer to fill out the questionnaire and scale. The study protocol was reviewed and approved by the internal human trial review board of Far Eastern Memorial Hospital.

Estimation of sample size was determined with G*Power 3.1 software, and multiple linear regression was used in the *F* test: In the fixed model, estimates were made using an *R*^2^ deviation from zero approach, with *α* set as .05, and power = 0.8; the effect size was conservatively estimated as 0.15. The sample consisted of 131 persons, and 14 additional subjects were included in anticipation of an invalid sample rate and dropout rate of approximately 10%; as a result, the total sample consisted of 145 persons. The interview rejection rate was 7.4% (10 patients), and the valid effective questionnaires' response rate was 100%. After 10 patients were excluded during interviews because of physical weakness or other personal reasons, and another 19 patients were excluded for incomplete questionnaires or dropping out, the final analytical sample was 126 patients.

### Instruments

#### BASIC INDIVIDUAL ATTRIBUTE DATA QUESTIONNAIRE

This questionnaire included demographic variables including age, gender, marital status, level of education, economic status, religious beliefs, medical history, and main caregivers. Medical variables included location of primary cancer, disease duration, time since surgery, frequency of treatments (frequency of surgery, radiotherapy, and/or chemotherapy), treatment times, and recurrence or not.

#### SHORT-FORM CANCER NEEDS QUESTIONNAIRE

This scale contains a total of 32 questions in 5 domains: psychological needs, health information needs, physical and daily living needs, patient care and support needs, and interpersonal/communication needs.^18–20^ The Short-Form Cancer Needs Questionnaire (CNQ-SF) has a 5-point scoring system; responses consist of no needs/not applicable, no needs/already met, low needs, moderate needs, and high needs; possible scores for each question range from 1 to 5 points, and total possible scores after adjustment range from 0 to 100 points, where a higher score indicates higher needs in a specific domain. After we obtained consent of the author^18^ for translation, the scale was translated into Chinese through a 2-stage process using foreign scale translation procedures. Two physicians specializing in oral cancer were invited to translate the original foreign-language scale into an initial Chinese version, and the translations of the 2 physicians were revised as the second Chinese version following a group discussion. Two individuals conversant in both Chinese and English were then asked to translate the second Chinese version back into English. It was then subjected to a group discussion to check for consistency with the original foreign language version, and 5 experts assessed the scale's correctness, clarity, and cultural differences affecting translation using a 5-point Likert scale (where 1 point indicated strong disagreement, and 5 points indicated strong agreement) to determine content validity index. The experts consisted of nurses, dentists, and oral surgeons. The final content validity index value was 0.9286, which was greater than 0.9, and indicated excellent content validity. The Chinese version achieved standard reliability with Cronbach *α* coefficient = .95. The original Chinese version (prior to evaluation for the present study) of the CNQ-SF was validated by Chen et al^21^ in oral cancer patients and was found to have (1) good internal consistency reliability for the overall scale and subscales; (2) good 1-week test-retest reliability (correlation = 0.80) for the overall scale; (3) construct validity, supported by 6 clearly identified factors explaining 74.87% of the variance; and (4) convergent validity, supported by correlations among its subscales and related scales, as well as by discriminating care needs according to undergoing versus not undergoing reconstructive surgery and cancer staging. European Organisation for Research and Treatment of Cancer Head and Neck Cancer Quality of Life Scale (EORTC QLQ-H&N35) is the most advisable scale to measure QOL in oral cancer patients.^22^ The questionnaire evaluates secondary symptoms and effects of the treatment, including smell, salivation, sensory affectation, speech, social eating, dental problems, oral opening limitations, sticky saliva, and others.^23^ The Chinese version of the scale was validated in Taiwan^24^ with Cronbach *α* coefficient > .7. This scale has a total of 35 questions grouped into 7 scales: pain (4 questions), swallowing (5 questions), senses (2 questions), speaking (3 questions), eating in the company of others (4 questions), social contacts (4 questions), sexuality (2 questions), and 11 individual questions concerning teeth problems. The first 30 items allow respondents to select on the basis of their level of symptoms. The answers have 4 levels: “none whatsoever,” “a few,” “some,” and “many,” which are scored from 1 to 4 points, respectively. The final 5 questions have the possible responses of “yes” and “no,” which are scored as 1 and 2 points, respectively. After adjustments, total possible scores range from 0 to 100 points, where higher scores in this module represent a higher level of symptoms and problems associated with cancer.^25^ The original Chinese version of EORTC QLQ-H&N35 (prior to evaluation for this study) was validated by Chie et al^26^ and found to have moderate to high test-retest reliability and high internal consistency in most scales and was able to show the expected differences between patients in active treatment and follow-up groups.

### Statistical Analysis

All statistical analyses were carried out using IBM SPSS statistical software version 22 for Windows (IBM Corp, Armonk, New York). Demographics, clinical characteristics, QOL scores, and care needs were summarized as mean ± SDs for continuous variables or n (%) for categorical variables. Pearson correlation analysis determined the coefficient of correlation (*r*). Univariate analysis was performed to identify associations between QOL or cancer needs and patients' demographics and clinical characteristics using Pearson correlation analysis for continuous variables; however, for categorical variables in patients' demographics and clinical characteristics, QOL scores and care needs were summarized as mean ± SD for given items and compared using either the *t* test or 1-way analysis of variance. A post hoc Bonferroni correction was then done. For variables with a significant association, stepwise multiple linear regression analysis was used to identify the factors associated with cancer needs. All variables that were significantly associated with cancer needs in univariate analysis (*P* < .05) were placed into multiple linear regression analysis. Results were shown as estimated *β* with corresponding SE and *P* values for each variable and *R*^2^ value for the prediction model. All statistical assessments were 2-tailed and considered statistically significant at *P* < .05.

## Results

### Patients' Demographic and Clinical Characteristics

A total of 126 patients (119 males/7 females) with a mean age of 57.6 years (range, 33–88 years) were enrolled in this study. Patients' demographic and clinical characteristics are summarized in Table 1. In terms of demographics, the majority of patients were married (73.8%), had an economic level of less than 20 000 new Taiwan dollars per month (67.5%), and were religious (83.3%), and most of their caregivers were family members (68.2%). In terms of clinical characteristics, 55 patients (43.7%) were in cancer stage IV; 53 (42.1%) had a disease duration of more than 2 years; 94 (74.6%) were within 12 months of surgery; 81 (64.3%) were within 6 months of their latest therapy; 71 (56.3%) had only surgical treatment; 69 (54.8%) had received radiation therapy; 66 (52.4%) had received chemotherapy; and 32 (25.4%) had experienced at least 1 recurrence (Table 1).

**Table 1:**
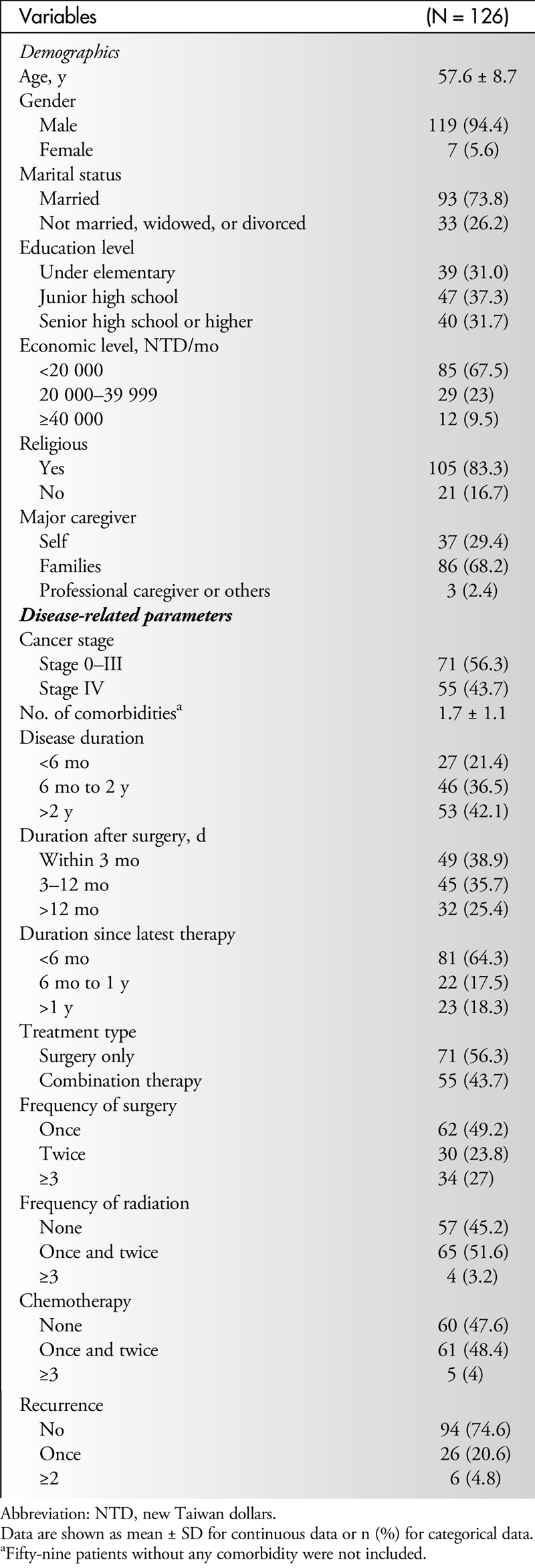
Subjects' Demographic and Clinical Characteristics

### HRQOL and Cancer Needs

Table 2 summarizes scores of the questionnaires, EORTC QLQ-H&N35 and CNQ-SF scales. The total scores were 28.81 ± 17.16 and 38.34 ± 20.85 for HRQOL and care needs, respectively. For the QLQ-H&N35, results showed that most of the patients were troubled by teeth problems, opening the mouth, and dry mouth, but less troubled by feeding tubes, weight gain/loss, and social contact. For the CNQ-SF, the highest and lowest scores were found in the “health information domain” and “interpersonal communication domain,” respectively.

**Table 2:**
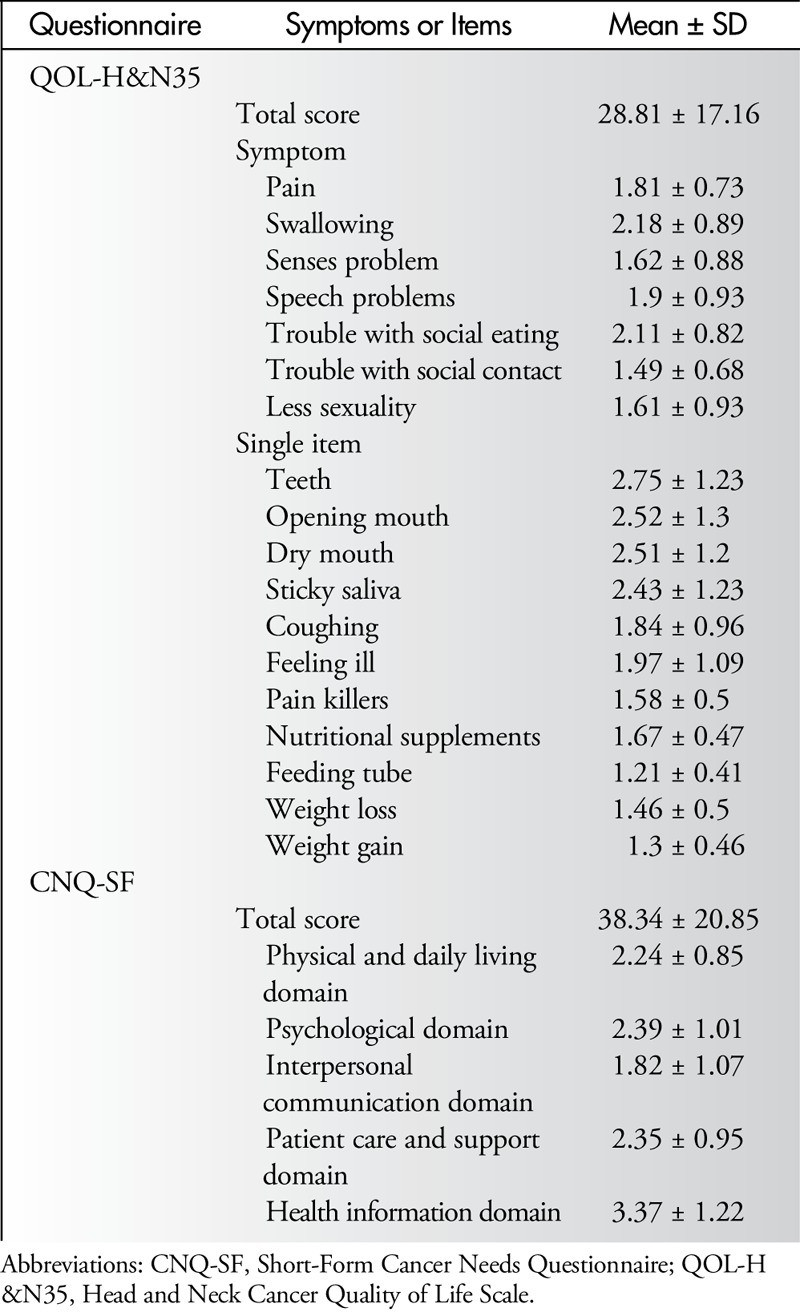
Summary of QOL-H&N35 and CNQ-SF Scores (N = 126)

### Factors Associated With Care Needs and HRQOL

Table 3 shows results of univariate analysis of associations between patients' characteristics and total scores of 2 questionnaires. Increases in CNQ-SF scores were associated with female patients, family as caregivers, having disease duration of more than 2 years, having treatments more than 3 times including surgery, chemotherapy and radiation, and experiencing recurrence (all *P* = .05). Increases in QLQ-H&N35 scores were associated with economic level and all of the disease-related parameters, except number of comorbid conditions (all *P* ≤ .041). All significant variables were placed into multiple regression analysis (Table 4). Results indicated that female patients and patients receiving 3 or more treatments of chemotherapy were associated with increased care needs according to increased CNQ-SF scores (*β* = 0.177, and 28.49; both *P* < .05), and patients receiving 3 or more treatments of chemotherapy were associated with QOL according to increased QLQ-H&N35 scores (*β* = 27.77, *P* = .007) (Table 4).

**Table 3:**
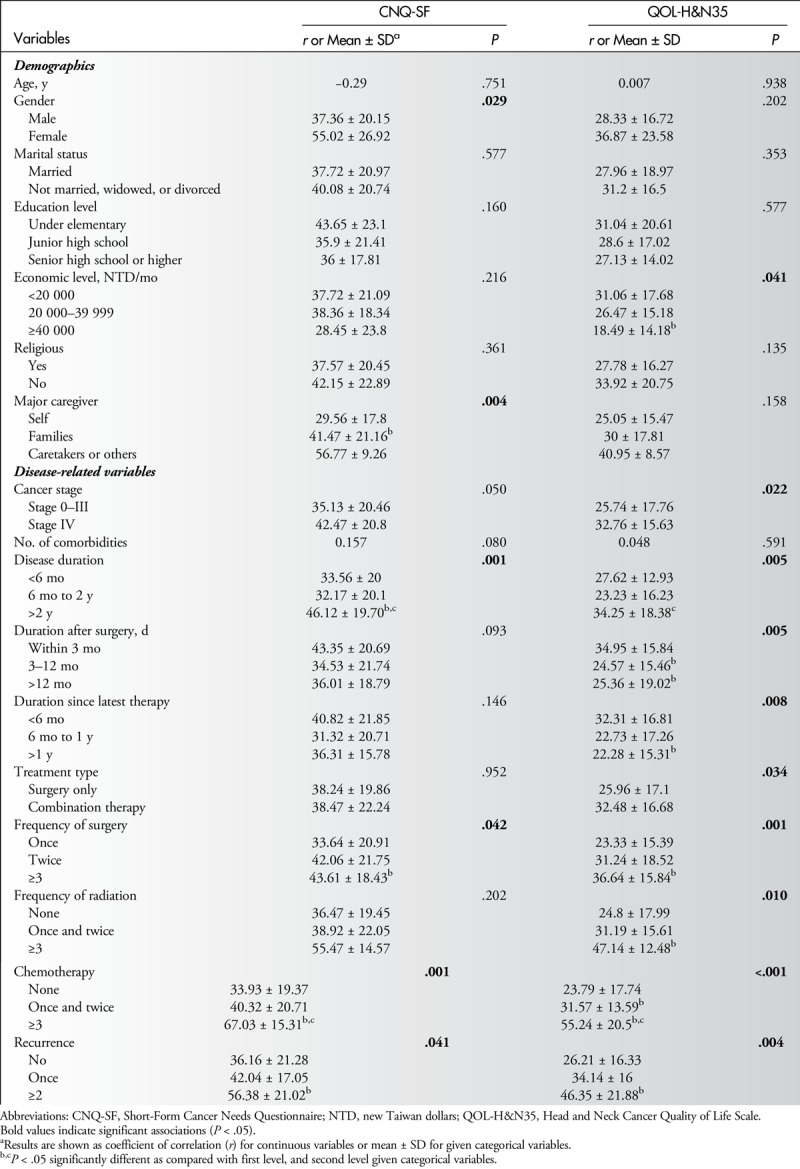
Univariate Analysis of Associations Between Patients' Characteristics and Total Scores of CNQ-SF and QOL-H&N35 (N = 126)

**Table 4:**
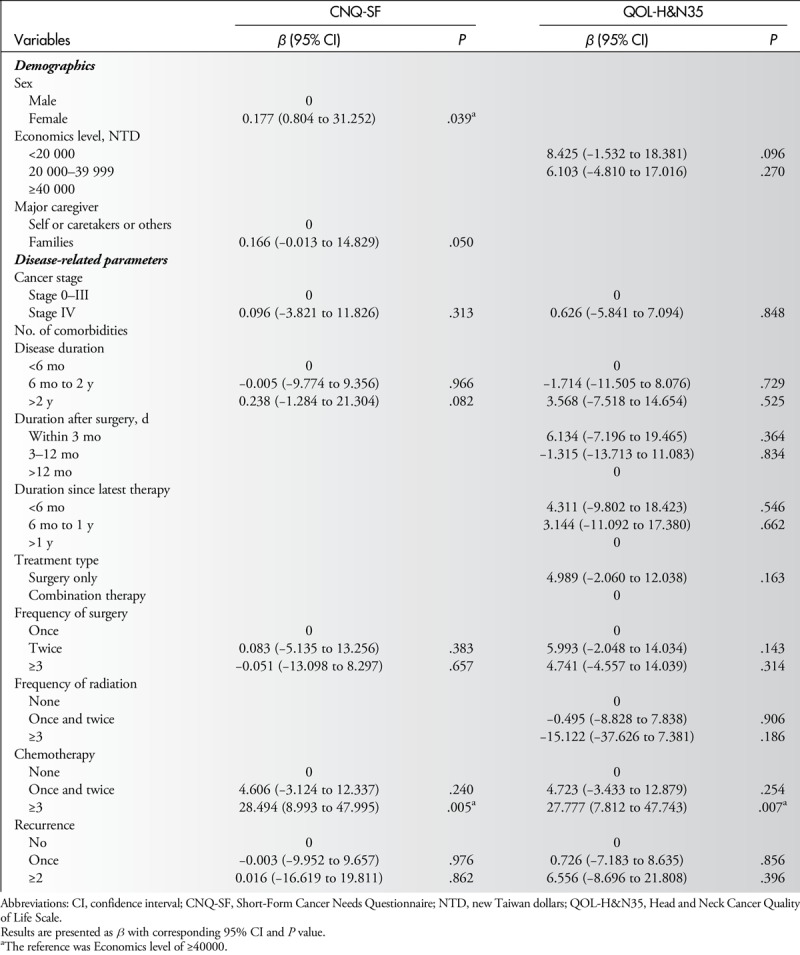
Multiple Linear Regression Analysis of Associations Between Patients' Characteristics and Total Scores of CNQ-SF and QOL-H&N35

### Correlations Between HRQOL and Cancer Care Needs

Table 5 shows that all HRQOL-related symptoms in QLQ-H&N35 correlated significantly with the total score of CNQ-SF. However, in correlations between individual domains of care needs, the “sense problem” in QLQ-H&N35 did not correlate with “psychological needs” and “needs of healthy information,” and “less sexuality” in QLQ-H&N35 did not correlate with “needs of healthy information” and “interpersonal communication.”

**Table 5:**
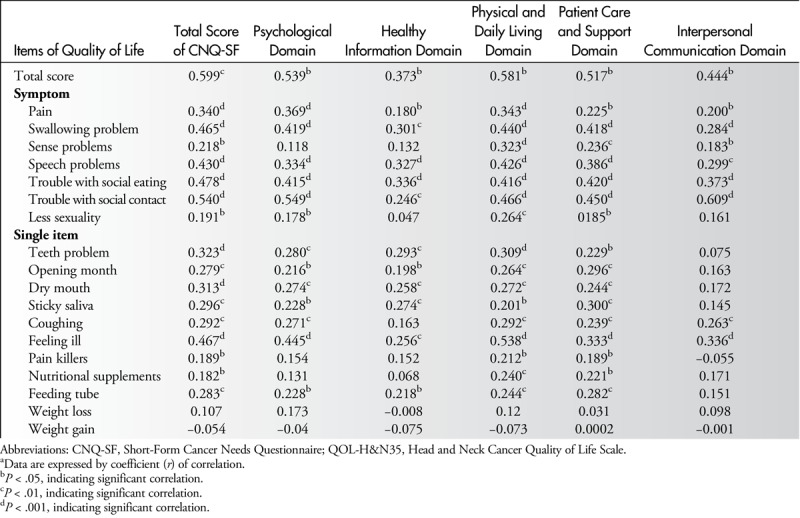
Correlation Analysis of QOL-H&N35 With CNQ-SF^a^

Most of the single items in QLQ-H&N35 correlated significantly with the total score of CNQ-SF, except for “weight loss” and “weight gain.” However, in correlations between individual domains of care needs, “pain killers” and “nutritional supplements” in QLQ-H&N35 did not correlate with “psychological needs.” “Coughing,” “pain killers,” and “nutritional supplements” in QLQ-H&N35 did not correlate with “need for healthy information.” In addition, only “coughing” and “feeling ill” in QLQ-H&N35 correlated significantly with the “need for interpersonal communication.”

All variables that were significantly associated with cancer needs in univariate analysis (*P* < .05) were placed into a stepwise multiple linear regression analysis (Table 6). Four HRQOL-related variables, “trouble with social contacts,” “swallowing problems,” “teeth problems,” and “feeling ill,” were significantly associated with higher care needs in oral cancer patients (all *P* ≤ .05).

**Table 6:**
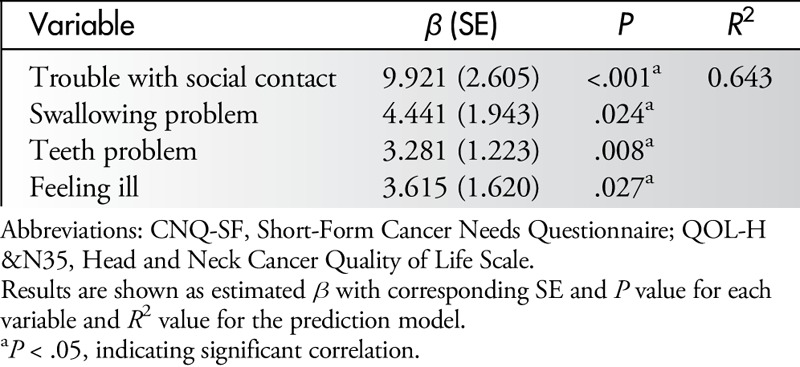
Multiple Linear Regression Analysis of Associations Between QOL-H&N35 Items and Total Scores of CNQ-SF (N = 126)

## Discussion

Although the major objective of this study was to investigate the possible correlation between HRQOL and care needs in oral cancer patients, a comprehensive assessment of HRQOL and care needs was performed before establishing that correlation. Multiple linear regression analysis revealed that demographic factors such as gender (female) and clinical factors such as chemotherapy were associated with care needs, and only chemotherapy was significantly associated with HRQOL after the multiple linear regression analysis was performed. A significant correlation between HRQOL and care needs was demonstrated. Results of univariate analysis indicated that almost all HRQOL-related symptoms and problems correlated significantly with the total score of care needs except for “weight loss” and “weight gain.” Stepwise multiple linear regression analysis specifically identified 4 variables: “trouble with social contacts,” “swallowing problems,” “teeth problems,” and “feeling ill” as the independent predictors of higher care needs in oral cancer patients.

The postoperative QOL of oral cancer patients is generally believed to hinge upon the course of treatment. A prospective study found that patients invariably had a poorer QOL after surgery, and QOL decreased dramatically during the first 3 months after surgical treatment.^27^ In that study, the reconstruction type was an independent factor that influenced QOL and functional results after free flap reconstruction, and the author concluded that the reconstructive techniques played a crucial role in maintaining a satisfactory QOL. In the present study, duration after surgery, treatment type, and frequency of surgery were associated with HRQOL, and even the frequency of radiation and chemotherapy was closely associated with HRQOL.

Malnutrition and loss of weight are major problems of patients with oral cancer after surgery, radiotherapy, and/or chemotherapy.^28^ Those authors suggested that malnutrition and loss of weight in these patients are usually a result of inadequate nutrition caused by the functional restrictions imposed by chewing and swallowing problems, and the mental problems of depression and related lack of appetite. Correlations have been shown between chewing and swallowing and mobility of the tongue, mobility of the mandible, and mouth opening.^27,29^ Patients with chewing and swallowing problems have a reduced QOL. The loss of teeth and the fit and stability of dental prostheses must also be considered. Results of the present study confirm that HRQOL-related problems such as teeth problems, dry mouth, opening the mouth, and sticky saliva are closely associated with the total score of the care needs questionnaire. In addition, swallowing problems and teeth problems are 2 independent factors that predict an increase in care needs among oral cancer patients. Nutritional supplements correlated significantly with the total score of the care needs questionnaire. Specifically, nutritional supplements correlated significantly with the needs related to physical and daily living as well as the needs for patient care and support. However, weight change, either weight loss or weight gain, did not correlate with the care needs of oral cancer patients.

A qualitative study has suggested that, with regard to the life experience of postoperative oral cancer patients, apart from the obvious impact on patients' mouths and suffering life-threatening symptoms, patients' QOL may also be affected by such factors as consciousness of their survival, restrictions on interpersonal relationships, state of adaptation, and establishment of a support network.^30^ Results of the present study support the previous finding that the psychological domain and the need for interpersonal communication of care needs correlated significantly with HRQOL-related symptoms. In addition, difficulties with social contact and feeling ill psychologically were 2 independent factors that predicted an increase in care needs among oral cancer patients. Several HRQOL-related problems, such as teeth problems, dry mouth, and opening the mouth, were also closely associated with psychological needs. However, the problems of pain killers and nutritional supplements did not correlate with psychological needs, and only the problems of coughing and feeling ill correlated significantly with the need for interpersonal communication.

In clinical practice, nursing care for oral cancer patients may benefit from use of the HRQOL questionnaire to evaluate the severity of postoperative symptoms while patients are recovering. In addition, the needs assessment scale can be used to evaluate the needs priorities of postoperative patients, also helping caregivers to identify the items with which patients are dissatisfied. These tools are readily available and may help nurses to optimize the quality of personal care and establish solid relationships with patients.

In the present study, HRQOL assessment indicated that solid food dysphagia, dental conditions, trismus, xerostomia, and social eating were the most prevalent items noted by patients and most deserving of attention from clinical caregivers. For example, patients can be given nutrition consultation and be trained to consume liquid diets. They could also be encouraged to maintain oral hygiene to avoid intraoral bacterial infection during inpatient care. Encouraging patients to join a support group may help to increase their disease-related knowledge and allow them to share their experiences with other patients. The support group may help to prevent or relieve social withdrawal and psychological disturbances associated with their illness. Results of HRQOL assessment also indicated that patients with lower incomes, long-standing illness, and recent postoperation/treatment, as well as end-stage patients and those with patients with numerous treatments and relapses, are especially in need of the attention and support of clinical caregivers to help improve QOL, postoperative recovery, and symptoms. The most highly scored items on the needs assessment scale were health information, mental/caring support, and doctor-patient communication. Therefore, in order to ensure that postoperative inpatients and outpatients are satisfied with the health information, psychological support, and care they receive, the care team of doctors, cancer case managers, psychologists, and social workers must work together with a positive supportive attitude to establish and maintain good relationships with the patients. Results of the HRQOL also showed that QOL is lowest during the first 3 months after surgery, whereas the average scores of needs assessment were the highest during the same 3 months. Therefore, 3 months after surgery appears to be the best time at which to execute interventional care.

Nursing education, in addition to advancing nurses' clinical knowledge and skills, should include the concepts of the HRQOL and the needs assessment scales. To achieve the goal of delivering comprehensive care, the 2 scales go beyond clinical care that focuses primarily on treating physical symptoms, allowing nurses to deal with different aspects of patients' needs. Findings of the present study suggest that patient education that provides health-related information is essential for postoperative oral cancer patients. The contents and skills in patient education must be optimized individually so that it is understandable and meets patients' needs.

The present study has several limitations. The cross-sectional research design used the 1-time questionnaire to investigate patients' QOL and care needs, which gained an understanding of postoperative oral cancer patients' care needs and QOL at only 1 particular point in time and did not allow evaluation of long-term changes. The use of purposeful sampling of patients at a single medical center in northern Taiwan reaches a conclusion only for that population and may not reach a generalized conclusion applicable to postoperative oral cancer patients nationwide. Finally, there is no specific HRQOL questionnaire for oral cancer symptoms and disorders such as mouth dryness, swallowing, chewing, eating, and talking. Within the questionnaires that measure QOL in oral cancer, only the EORTC Quality of Life Questionnaire Core 30 and an additional specific module for head and neck cancer (EORTC QLQ-H&N35) were used for oral cancer patients in previous studies, although it has been used along with the EORTC Quality of Life Questionnaire Core 30 in many large multicultural studies mainly in Nordic countries.^22^ However, there are differences in physiological function (eg, swallowing, opening one's mouth, sense of taste, and language impairment) between head and neck cancer and oral cancer, and the mode of soft-tissue reconstruction has the most profound impact on QOL after ablative surgery for oral cancer.^31^

In conclusion, a significant correlation exists between postoperative oral cancer patients' HRQOL and their care needs, suggesting that patients' care needs will increase as their satisfaction with their QOL decreases. Four oral cancer–related symptoms and problems, “trouble with social contacts,” “swallowing problems,” “teeth problems,” and “feeling ill,” are independent predictors for higher care needs in oral cancer patients. Using a valid HRQOL scale and a nursing needs assessment scale may help nurses gain an understanding of the severity of symptoms in postoperative oral cancer patients and determine their perceived physical and psychological care needs. Study results may also be useful in guiding the development of QOL measures specific to oral cancer patients.

Further research is warranted to explore HRQOL and needs assessment for different ethnic groups and diseases. In addition, based on results of the present study, interventional studies may be conducted to assess whether information about patients' HRQOL and care needs can be used effectively to help patients interact with family and society after oral cancer treatment and recovery.
